# CREAM, a component level coffeemaker electrical activity measurement dataset

**DOI:** 10.1038/s41597-020-00767-w

**Published:** 2020-12-17

**Authors:** Daniel Jorde, Thomas Kriechbaumer, Tim Berger, Stefan Zitzlsperger, Hans-Arno Jacobsen

**Affiliations:** Department of Computer Science, Chair for Application and Middleware Systems, Technical University of Munich, 85748 Garching, Germany

**Keywords:** Energy management, Industry

## Abstract

Monitoring the internal conditions of a machine is essential to increase its production efficiency and to reduce energy waste. Non-intrusive condition monitoring techniques, such as analysing electrical signals, provide insights by disaggregating a composite signal of a machine as a whole into the individual components to determine their states. Developing and evaluating new algorithms for condition monitoring and maintenance-related analysis tasks require a fully-labelled dataset for a machine, which comprises standard industrial components that are triggered following a typical manufacturing process to produce goods. For this purpose, we introduce CREAM, a component level electrical measurement dataset for two industrial-grade coffeemakers, simulating industrial processes. The dataset contains continuous voltage and current measurements provided at 6400 samples per second, as well as the product and maintenance-related event labels, such as 370600 expert-labelled component-level electrical events, 1734 product ones and 3646 maintenance ones. CREAM provides fully-labelled ground-truth to establish a benchmark and comparative studies of manufacturing-related analysis in a controlled and transparent environment.

## Abstract (summary)

**Table:** 

Measurement(s)	electrical current • Voltage
Technology Type(s)	data acquisition
Factor Type(s)	type of industrial-grade coffeemaker • time series data

Machine-accessible metadata file describing the reported data: 10.6084/m9.figshare.13260029

## Background & Summary

Recent advances in artificial intelligence and the increasing implementation of modern cyber-physical systems in the manufacturing industry constitute the backbone of a new industrial revolution^1^. The monitoring of current conditions and internal states of industrial machines is fundamental to increase the production and energy efficiency^2^. The placement of sensors, to obtain detailed information about the behaviour of the machine’s individual components, is fundamental in the condition monitoring (CM) process^2^. Instead of intrusively measuring each component of a machine individually, an aggregated signal for multiple components can be considered. In a subsequent step, algorithms to extract the per-component information from an aggregate signal can be applied. Such an approach can allow for avoiding invasive interference that causes various problems, such as high costs associated with sensor implementation and warranty issues. Initially developed to provide feedback on energy consumption in residential environments, non-intrusive load monitoring (NILM) is widely used for other purposes, such as CM^3,4^. NILM algorithms can be used to disaggregate power signals measured at the electrical mains of a building into the individual appliances^5,6^. By implementing sensors, such as Hall Effect current ones, electrical signals of a machine or appliance can be measured in a non-intrusive manner^4^. Sampling the voltage and current signals at high rates is necessary to identify individual components when many other components are concurrently activated and to enable differentiation between smaller ones^7^. To the best of our knowledge, there are two public datasets containing the data on electrical measurements of industrial-like machinery. Both of them have drawbacks, such as being either sampled at low rates^8^ or comprising only individual appliances from a laboratory environment^9^. The first dataset contains electrical parameters of a poultry feed factory, recorded for a duration of 111 days. The smart meters at the factory sample the data internally at 8000 sps, but send out the down-sampled electrical features once per second. This dataset provides insights into the energy consumption of a factory using NILM techniques for energy disaggregation. The machine components in this factory produce pellets of ration for poultry by processing corn or soybeans. The dataset comprises two pelletisers, two double-pole contactors, two exhaust fans and two milling machines. All appliances measured are horizontal motors^8^. In the second dataset, electrical signals for fifteen residential and industrial electrical components were sampled at 50000 sps in a laboratory environment. However, the utilised devices were not activated according to a dedicated pattern, for example, such as a production process, and no complementary information about conditions of components is provided^9^. In addition to these two datasets, several other datasets containing sensor measurements for CM concerning individual components were established^10–12^. These datasets contain information about the isolated components using a dedicated sensor infrastructure to obtain various parameters. The milling dataset by Agogino and Goebel^10^, for example, provides records on the wear of the milling insert of a milling machine, recorded at different speeds, feeds and depth of cut^10^. Some of the datasets provide additional information about detected faults of components, such as, for example, a hydraulic test rig^12^. In particular, in this dataset, measurements on the condition of hydraulic components in a primary working and a secondary cooling-filtration circuit are presented^12^. The sensor data includes features such as, for example, pressure, motor power, temperature, and vibration, measured at least once per second. The dataset includes component-specific failure information. The failure information for each component is structured hierarchically, from full functionality to failure of the component^12^.

To construct a dataset that would enable the evaluation of algorithms for non-invasive CM, event detection, and other manufacturing-related analysis tasks, we formulated the following requirements. First, a considered machine had to execute an industrial process, including typical electrical components that are used in manufacturing, triggered following dedicated process patterns. Second, the environment and the machine had to be fully-controllable to avoid any unknown external interference. Third, the machine had to be equipped with sensors to record reliable ground-truth for events caused by components. We focused on the events related to the fabricated products and performed maintenance actions. Following these requirements, we selected two distinct fully-automated, industrial-level coffeemakers to construct the proposed coffeemaker electrical activity measurement (CREAM) dataset, and to enable individual machine analysis and comparative studies between the coffeemakers. We provide high-resolution continuous measurements of the voltage and current signals of the coffeemakers acquired at 6400 sps. During signal acquisition, the machines produced eight different product types, each following a unique internal process. Furthermore, we provide 370600 expert-labelled electrical events, triggered by the machine components. In addition, CREAM contains the labels for the three main components of the coffeemakers, namely the respective heaters, pumps, and motors of the milling plants. The data are marked with the product and maintenance labels, containing the information about the fabricated products and performed maintenance actions. Therefore, the resulting dataset can be considered as a source for a wide variety of tasks, such as CM, product analysis, and maintenance prediction.

## Methods

**Fig. 1:**
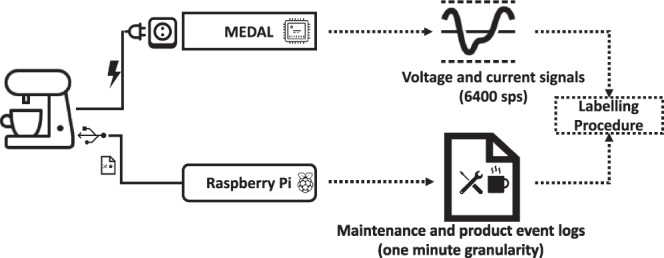
Data collection architecture. The setup consists of the MEDAL unit for measuring the voltage and current signals and the Raspberry Pi for pulling the maintenance and product event logs with a one-minute resolution from the serial ports of the coffeemakers. Afterwards, we applied the three-step labelling procedure, as shown in Fig. 2.

We constructed the CREAM dataset based on the previously defined requirements. For the *Jura GIGA X8*, the information accumulated in the dataset was recorded for a period from 23 August 2018 to 8 October 2018. We recorded the *Jura GIGA X8* dataset for sixteen hours every day, except for the last one, 8 October 2018, that was measured for eight hours. The data for the *Jura GIGA X9* was recorded for $20$ days, starting from 22 December 2018, for 15 hours per day. The daily data acquisition time frames were chosen to cover the main periods the coffeemakers were activated. For both coffeemakers, the data acquisition process was divided into three sub-steps, that apply equally to both machines. The data acquisition setup is shown in Fig. 1. First, we sampled the voltage and current signals of each coffeemaker at 6400 sps. To execute this step, we utilised a custom measurement device for high-sampling rate plug-level appliance recordings, namely, the mobile energy data acquisition laboratory (MEDAL) measurement unit^13^. Simultaneously, we extracted the product and maintenance-related event logs from a serial port of the coffeemaker using a single-board personal computer (PC). As the methodology described in the following is meant to apply generically to other scenarios, we refer to the types of coffee the coffeemakers produce as products. Lastly, three experts labelled the electrical component events and refined the automatically generated product and maintenance logs. In the next section, we provide general information about the considered coffeemakers, such as their individual components, the production processes, and other relevant characteristics.

### Domain knowledge

We measured the voltage and current consumed by two different professional coffeemakers, the *Jura Giga X8 Professional*^14^ and the *Jura Giga X9 Professional*, and combined them with the hand-labelled components and machine-generated event-logs. Below, we describe the domain knowledge and architecture useful to interpret the generated data, based on the official technical description and the components of the machines. Concerning the general production approach, pre-defined processes trigger the individual components of the machine for brewing a requested coffee product. First, a grinder is launched to grind pre-roasted beans and feeds the ground coffee to the brewing unit. Second, if not already pre-heated, heating units are utilised to heat the water or to produce steam for the requested coffee product. The water or steam is then pressed through the brewing unit, which controls the water flow through the ground coffee. A dedicated steam or a water pump moves steam or water through the machine. Then, the brewing unit presses water through the coffee to extract the ingredients, such as caffeine and oils, from coffee. After the brewing unit, the brewed coffee flows into a drinking container and is output from the coffeemaker. The brewing unit then pushes the residual coffee into the coffee tray. Depending on a particular coffee product, intermediary steps such as heating milk and producing milk foam, are executed. The *Jura Giga X8* has two thermal heating blocks. Therefore, steam required to produce milk foam and hot water is generated simultaneously. In contrast, the *Jura Giga X9* has one additional thermal heating block and an additional pump, to speed up the production process, especially for hot water^15^. In addition to the described brewing process, the coffeemakers have other maintenance programmes to ensure the long-term functionality of the machines and to speed-up the brewing process. These maintenance processes involve various actions, such as, for example, regular cleaning and descaling the coffee and milk systems.

Each coffeemaker is comprised of several major components and a variety of small ones. The main components involved in the production process are pumps, thermal heating blocks, and ceramic grinding modules, as listed in Table 1. The selection of the main components was performed according to the feasibility of detecting them visually in the electrical signal by the human experts. Therefore, the other components included in the coffeemakers, such as, for example, lights, valves, a touchscreen, and a drainage motor, were excluded from consideration due to their small power consumption or more complex power usage patterns.

**Table 1:** Main components of the coffeemaker.

Component	X8	X9	Characteristics	Power
water pump	1x	2x	15 Bar pressure	65 W
steam pump	1x	1x	15 Bar	28 W
thermal heater	2x	3x	—	1080 W
grinding motor	2x	2x	DC motor	26 W–236 W

The list outlines the main components, key characteristics, and their energy consumption.

The *Jura GIGA X8* is composed of two grinders, one for espresso beans and one for coffee ones, launched depending on a requested product. Each of the grinders is powered by a directed current (DC) motor. The motor energy usage depends on the speed it is running at. Therefore, its power consumption is within a specific range, as outlined in Table 1. Furthermore, thermal heating blocks are employed to produce hot water and steam when the machine generates a product or when the built-in pre-heating controller launches the heating process. In this way, the coffee-making process is sped up, as heating water to the required temperature is time-consuming. Hot water and steam are transported through the machine using the corresponding pumps. At the end of the process, water is pressed through the brewing unit. The timing and energy consumption for these components varied according to particular products and settings of the machine. As previously mentioned, the *Jura GIGA X9* has three thermal heating blocks and three pumps to speed up the production process.

The entire *Jura Giga X8* coffeemaker has a nominal capacity of 2700 W and a standby power consumption of approximately 0.5 W when operating it at the base-frequency of 50 Hz^14^. The *Jura Giga X9* differs, as is has a nominal capacity of 2300 W, while having the same standby power consumption^15^.

### Voltage and current monitoring

A single MEDAL measurement unit was used to collect the voltage and current signals^13^. MEDAL comprises an off-the-shelf power strip, a voltage, and a current sensor, as well as an embedded single-board PC for processing recorded measurements. The MEDAL system was initially developed to record a long-term office environment dataset for energy disaggregation^16^. Therefore, it complies with the high-requirements concerning data quality and long-term continuous recording. Each MEDAL unit has six sockets available, enabling it to measure six devices simultaneously. The data for each coffeemaker was collected independently and sequentially. Hence, we describe the setup exemplary for one of the coffeemakers in the following. We used two sockets to monitor the coffeemaker. The coffeemaker was plugged-into one socket (socket 1), and the other socket (socket 6) was used to record the background-noise generated by the measurement device. In this way, we facilitate noise filtering for users. Socket 1 was explicitly designed for measuring high-power devices (up to 3600 W). In the case of exceeding this limit, the recorded signal is limited to the maximum value, while keeping the operation electrically safe. The measurement unit itself consumes 5 W.

A hall effect-based sensor from the *Allegro ACS712* family recorded an independent current signal for each of the sockets. Furthermore, one voltage signal was recorded for each coffeemaker. MEDAL’s sampler board is used to digitise the analogue signal and to transmit the data via USB connection to the single-board PC, a Raspberry Pi 3. Here, seven independent single-channel analogue digital converters (ADC) *MCP3201* with a 12-bit resolution are used^17^. Despite utilising independent ADCs, MEDAL samples the signals simultaneously, coordinated by an *ATmega324PA* microcontroller. The recorded data were stored on a SSD hard-drive connected to MEDAL via USB.

MEDAL is capable of recording the signals with a high temporal resolution without introducing data losses and gaps, which allows capturing the voltage and current signals at 6400 sps. These high sampling rates enable extracting the frequency-domain related features for various analytical purposes^7^.

### Product and maintenance events

In addition to recording electrical signals, we collected the product and maintenance event logs that were automatically generated by the coffeemakers and read out over the serial maintenance ports of the machines. We used the setup and the information described in the coffeemaker reengineering project repository and documentation provided by the company Q42^18^. For each coffeemaker, a Raspberry Pi microcontroller was connected to the serial maintenance port, using its receiver and transmitter pins to establish an 8-N-1 serial connection. Then, the events were extracted from each coffeemaker’s internal EEPROM using the reverse-engineered codes provided in the repository and stored on the SSD hard-drive. The raw events generated by the machines were marked by timestamps with a one-minute time resolution and were created after or close to the completion of an event.

While measuring electrical signals, eight different products were produced by the two coffeemakers. In addition to producing these products, the coffeemakers were capable of providing a wide variety of other hot water and milk-based products. The products mentioned in the dataset are listed in Table 2. We omitted the products that were not produced when data collection was enabled.

**Table 2:** The list of fabricated products of both coffeemakers.

Name	Milling Plant	Milk	Two X8 | X9
cappuccino	espresso	Yes	Yes | Yes
coffee	coffee	No	Yes | Yes
espresso	espresso	No	Yes | Yes
hot water	—	—	—
latte macchiato	espresso	Yes	Yes | Yes
white coffee	espresso	Yes	Yes | −
ristretto	espresso	No	Yes | Yes
espresso macchiato	espresso	Yes	Yes | Yes

The products have different production processes depending on the involvement of a type of a milling plant and the usage of milk. Some products can be produced simultaneously, as indicated by the column *Two* for both coffeemakers respectively.

The product considered indicates which components were utilised during the preparations process. When attempting to separate the behaviour of components that are built-in into the coffeemakers multiple times, such as grinding modules, the product information was analysed to identify the particular component involved. In addition to the product events listed in Table 2, we also recorded maintenance-related events, as listed in Table 3. Certain events were triggered to request a user to perform maintenance activities, such as, for example, to rinse the milk system. Other events refereed to the executed action, such as, for example, the machines rinsing the milk system. The *Type* column in Table 3 indicates whether an event is an alert for action (type*P*) or an action executed by the machines (type *A*). Both event types could be considered to extract and predict the maintenance-related information from the electrical data, as they described the current state of the system.

**Table 3:** Maintenance-related events of both coffeemakers.

Name	Description	Type
MillingPlantEspresso	Grinding espresso beans	A
MillingPlantCoffee	Grinding coffee beans	A
CleanMilkSystem	Cleaning the milk system	P
RinseMilkSystem	Rinsing the milk system	A
Time2Clean	Alert: Clean the coffee system	P
RinseCoffeeSystem	Rinsing the coffee system	A
Clean	Clean the whole system	A/P
Time2Descale	Descale the whole system	A/P

The list represents all maintenance-related events in the dataset and their purpose.

To illustrate this, we consider the following example. When rinsing milk or the coffee system, water is pumped through the respective pipes to remove the remains of the coffee making process. The *RinseMilkSystem* and *RinseCoffeeSystem* activities can be launched either automatically by the coffee maker or manually by a user after the *CleanMilkSystem* or *Time2Clean* alerts appeared on the screen. In contrast to using water for rinsing the system, the *CleanMilkSystem* alert requests a user to insert a cleaning agent into the machine. The standard procedure is to perform this task daily. The *Clean* alert requires the following actions from the user: the drip tray and the ground coffee container have to be removed and emptied. Then, a cleaning agent has to be used to clean the whole system. The coffeemakers can not be used before the completion of the cleaning process, which takes approximately 20 minutes. Similarly, the *Time2Descale* alert requests a user to add a descaling tablet into the water and to run the descaling programme that takes approximately 50 minutes^14,15^.

### Labelling procedure

The behaviour of electrical components was captured in the voltage and current signals recorded by the MEDAL unit. The electrical signals were marked according to three sets of labels aiming to facilitate a wide variety of supervised and unsupervised analysis techniques. We have defined an electrical event for both coffeemakers individually, based on the key characteristics the acquired signals exhibit. For the *Jura GIGA X8*, an electrical event was defined as an increase in the current signal equal to approximately one ampere that lasted over a time frame of at least 1 s. In order to capture all significant events, there can be slight deviations from the event definition, as the data exhibits some variation that we also captured in the labelling process. In contrast, the *Jura GIGA X9* generated a vast amount of patterns with a shorter duration. Hence, to capture this behaviour, we have created two sets of electrical event labels for the *Jura GIGA X9*. The first set contains electrical events lasting over a time frame of at least 1 s, similar to the component events of the *Jura GIGA X8*, to enable comparative studies with the other coffeemaker. The second set of component events of the *Jura GIGA X9* extends the first one with events lasting at least 0.1 s. Thus, we have labelled 92449 electrical events for the *Jura GIGA X8*. For the second coffeemaker, we have created 278151 electrical events, including the 44219 events labelled with a minimum duration of 1 s.

**Fig. 2:**
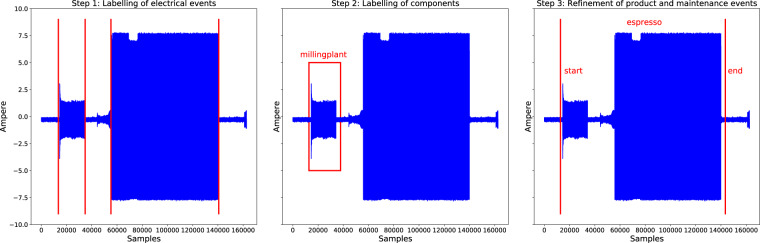
Three-step labelling procedure. The red, vertical lines denote the labels added at the respective step. In Step 1, the electrical events are labelled. In Step 2, events are assigned to the respective initiating component. In Step 3, the product and maintenance events registered with the one-minute granularity are precisely allocated in time.

**Fig. 3:**
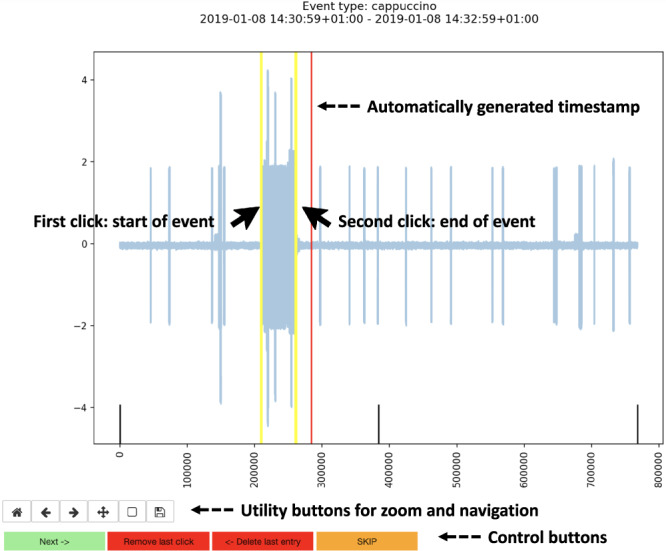
Labelling tool for step 3 of the labelling procedure.

Among all registered electrical events, we created a subset that contained the expert-labelled information about the individual main components that had triggered these events. Furthermore, the two sets of the maintenance and product-related events that were automatically generated by the coffeemakers were specified for each of the coffeemakers individually. Due to the aforementioned granularity of one minute, these events were manually refined to match the associated electrical signals as precisely as possible. The three sets of labels were constructed by applying a three-step labelling procedure conducted by three human experts, as outlined in Fig. 2. The experts involved in the labelling procedure own a university degree in computer science and have vast experience in signal processing and machine learning, making them suitable for the task. We have ensured a consistent labelling of events by definition the key characteristics, as explained above, and by using example events from the data to guide the experts. The labelling tools allow for high precision labelling of the time series data, as shown in Fig. 3. To reduce human labelling bias and to reduce errors, all events were peer-reviewed.

In the first step, we hand-labelled the electrical events that were triggered by the main electricity consumers in the coffeemaker. For this purpose, we developed a labelling tool, that enabled the experts to inspect signal segments and mark potential events visually. The labelling procedure was established according to the previously stated event definition. Furthermore, an event had to exhibit a significant and re-emerging pattern. After completion of the labelling procedure by two of the experts, all generated labels were revised and corrected by the third expert. The vast amount of events could be used to develop and benchmark event detection algorithms on a high number of samples, in contrast to the existing datasets.

In a second step, we assigned a subset of the labelled events to the corresponding main component, as listed in Table 1, that had caused these events to occur. In this way, we aimed to facilitate the development of supervised machine learning algorithms requiring prior labelling to identify main components in the coffeemakers. Two of the main components, grinding motors and thermal heating blocks, are installed multiple times in the coffeemakers. In the labelling process, we were unable to distinguish the components of the same type visually certainly. Therefore, we summarised the main components from Table 1 according to the three following classes: *heater*, *millingplant*, and *pump*. The precision ceramic disc grinding modules of the milling plants are powered by one motor each. The signals corresponding to these demonstrate a characteristically sharp spike after being switched on when a motor is initially accelerated. Afterwards, the amplitude slowly decreases when the motors settle into their steady-state. The grinder motors are the most prominent components in the coffeemakers, being clearly visible in the current signal. We considered the *MillingPlantEspresso* and *MillingPlantCoffee* events from the maintenance events list to obtain isolated milling events for the labelling process. After selecting a random subset from these events, the human experts manually labelled it using the labelling tool. For the *heater* events, we used the signals recorded on Saturdays. On these days, no products were generated, and no maintenance tasks were executed by the machines, as the locations where the coffeemakers were placed at were not occupied during weekends. Despite that, the machines were not switched off completely, and the installed pre-heating system periodically initiated the heating procedure to remain prepared for future provisioning. Therefore, we could observe the isolated heating events on these days, which facilitated labelling a larger sample of heater signals for the ground-truth. The labelling of pumps was performed using the *hot water* product events, as they involved no usage of grinders that infer with the pumping process. The *heater* components involved in the *hot water* process could be visually separated by the experts, as they steadily consumed the same amount of energy.

In the last step of the labelling procedure, depicted in Fig. 3, we manually refined the automatically generated product and maintenance event timestamps. The machine-generated timestamps had a one-minute resolution in time and marked the completion of a given procedure. Therefore, we plotted the signals enhanced with the labels from Step 2. The human experts then manually specified the start and end timestamps for a considered event by investigating the signal in the window of interest around the automatically generated timestamp. All labelling tools are available in the provided repository^19^.

### Known issues

The signals measured using the MEDAL system may introduce a slight direct current bias, occurring due to changes in the DC reference voltage and the use of a unipolar ADC. Appropriate signal calibration and filtering, as shown in the CREAM repository^19^, should be applied to correct this issue during pre-processing^16^. Furthermore, the events represented in the CREAM dataset are imbalanced, as shown in Table 4 for the *Jura GIGA X8* and in Table 5 for the *Jura GIGA X9*. When evaluating the performance of algorithms on the dataset, it is necessary to adjust for this bias by applying appropriate techniques for the imbalanced data, such as oversampling.

**Table 4:** Statistics of the product and maintenance event durations of the *Jura GIGA X8*.

Event type	Samples	Mean	Standard deviation
cappuccino	521	50.26	3.51
coffee	361	29.43	3.24
espresso	313	23.43	3.08
hot water	157	24.16	6.33
latte macchiato	109	46.10	8.40
white coffee	10	29.86	0.94
ristretto	3	21.29	1.99
espresso macchiato	2	37.79	1.06
MillingPlantEspresso	1316	4.53	1.65
MillingPlantCoffee	1008	4.99	1.93
RinseMilkSystem	418	17.04	2.01
CleanMilkSystem	47	49.44	6.86
Time2Clean	47	2.56	7.67
RinseCoffeeSystem	22	76.50	28.54
Clean	9	106.75	10.51
Time2Descale	1	0.53	0

The list shows the mean and standard deviation of event durations of the product and maintenance events produced by the *Jura GIGA X8*.

**Table 5:** Statistics of the product and maintenance event durations of the *Jura GIGA X9*.

Event type	Samples	Mean	Standard deviation
cappuccino	95	36.20	20.10
espresso	58	18.26	8.54
coffee	47	24.91	12.12
hot water	43	47.19	57.52
latte macchiato	14	53.68	41.13
espresso macchiato	1	36.19	0
MillingPlantEspresso	392	2.40	1.12
MillingPlantCoffee	209	2.34	1.03
RinseMilkSystem	117	8.73	11.14
CleanMilkSystem	20	24.08	23.34
Time2Descale	15	1.28	1.02
Time2Clean	14	1.03	0.82
RinseCoffeeSystem	8	15.70	16.394
Clean	3	154.00	55.31

The list shows the mean and standard deviation of event durations of the product and maintenance events produced by the *Jura GIGA X9*.

In addition, it should be noted that due to customisation possibilities and due to unexpected user behaviour, such as aborting the coffee-making process, the event durations may vary, as shown in Tables 4 and 5. This heterogeneity needs to be considered in the analysis, as the intra-class variance is high; namely, the signals for samples corresponding to the same type of event can deviate between each other.

The obtained voltage and current signals acquired are the aggregate ones corresponding to the individual component activities. Therefore, in the analysis, it is necessary to consider overlapping activities, such as heating and activating a milling plant.

## Data Records

The CREAM^19^ dataset contains the three measured signals generated by each of the coffeemakers: the voltage, current and background-noise signal registered by a socket in the MEDAL measurement unit. Furthermore, it comprises the labels of electrical components, as well as the information about the product and maintenance events. The dataset is divided into two subfolders, one for the *Jura GIGA X8* and one for the *Jura GIGA X9*, respectively.

### Data files

All signals were sampled with 6400 samples per second at the mains frequency of 50 Hz. The signals obtained from the sensor input were stored as-is: in particular, no dedicated pre-processing of the raw signals was performed to ensure unbiased analysis of the data. In the CREAM repository, we provide examples of possible pre-processing steps^19^. The dataset was structured with respect to the individual days of recording so that one subfolder contains the data files for each day in the data acquisition process. The raw data and the metadata were stored in HDF5 files. The utilised data formats and the metadata are similar to the ones used in the BLOND office environment dataset, as the MEDAL hardware was used in the latter as well.

Functionality to process this type of file is available in a variety of open-source and commercially available tools, making them easily accessible^16^. Into each of the HDF5 files, we embedded the corresponding file metadata in the form of HDF5 attributes that could be accessed either directly in the file root or in a specific HDF5-dataset, as described in Table 6. The value types of the data are either short integer, floating point or ASCII-encoded byte strings. Parts of the metadata information is also encoded in the file names, for example, *coffee-maker-2018-08-23T07-00-03.783395 T* + *0200-0000001.hdf5*: The first sample of this file was recorded approximately at 07:00 23 October 2018, with a time zone offset of 2 hours. Furthermore, each file within a day has a sequence number, such as the sequence number 1, as represented in the example file name. The sequence number uniquely identifies the file order within a particular day. All timestamps in CREAM, in particular, the ones from the labels and from the data recordings, are synchronised. In the CREAM repository, we provide the examples for handling timestamps and time zone information^19^. Each HDF5 file contains one hour of data, and each day of CREAM, except the last one, contains sixteen HDF5 files, with the first file starting at approximately 06:00, and the last file ending at 22:00, covering the usual working hours. No daylight saving time transitions or leap seconds have occurred during the process of recording. Therefore, one can fully rely on the timestamps provided in the data. The MEDAL units automatically create the one hour file chunks, while measuring the electrical signals without interruptions at 6400 sps.

**Table 6:** HDF5 file metadata.

Path	Attribute	Description
/	name	Name of the measurement unit
/	first trigger id	Internal trigger number to detect gaps
/	last trigger id	Internal trigger number to detect gaps
/	sequence	day-internal sequence number
/	frequency	nominal samples per second
/	year	Year of this file
/	month	Month of this file
/	day	Day of this file
/	hours	Hours of first sample
/	minutes	Minutes of first sample
/	seconds	Seconds of first sample
/	microseconds	Microseconds of first sample
/	timezone	Timezone offset
/<dataset>	calibration factor	Factor for signal calibration
/<dataset>	removed offset	Removed DC-offset

The metadata attributes are accessible via a HDF5-attribute-path. All physical values are provided in base units (Volt, Ampere, Hertz), and the timestamp information refers to the first sample in the respective data file. The < *dataset > *placeholder can be either *voltage*, *current1* for the coffeemaker’s current from socket 1, or *current6* for the socket 6 background-noise current.

### Labels

The labels resulting from the labelling procedure represented in Fig. 2 are stored as comma-separated value (csv) files in the sub folder of the respective coffeemaker. All label timestamps have the following format: *year*-*month*-*day hours*:*minutes*:*seconds*.*microseconds* + *timezone*. The electrical component events are stored in the *component events.csv* file for the *Jura GIGA X8*, as described in Table 7. In contrast, there are two component event files in the *Jura GIGA X9* subfolder, one for the previously defined minimum duration of the electrical events. The 1 s events are stored in the *component events coarse* CSV file and the 0.1 s events in the *component events fine* CSV file. The fine-grained events of the *Jura GIGA X9* can be matched with the corresponding coarse events, using the *ID* column of the label files. The events are either turn-on (*On*) or switch-off (*Off*) events. The *On* / *Off* information was determined automatically, by comparing the mean power in a 0.1 s window before the event and 0.1 s after the event occurs. If the mean power before the event is lower than afterwards, we labelled the event to be an *On* event. On the other hand, *Off* events exhibit a drop in power in between the pre-event and the post-event window. As stated before, we assigned one of the three components (heater, millingplant, or pump) to a subset of the events. The events without a component label are declared as *unlabeled* in the respective column.

**Table 7:** Description of the component events files.

Column	Description
Filename	File name containing the event
Timestamp	Event timestamp
Amplitude	Current value (ampere) of event
Event Type	*On* or *Off* event
ID	Unique event identifier, sequentially numbered
Component	Name of event invoking component

Columns of the files containing the electrical component timestamps and supplementary information, such as the amplitude of the current drawn.

The refined product and maintenance events are stored in the respective*.csv* files. These files have the same column structure, as shown in Table 8.

**Table 8:** Description of the product and maintenance event files.

Column	Description
Start Timestamp	Start time of event
End Timestamp	End time of event
Automatic Timestamp	Original automatically generated timestamp
Event Type	Product name or maintenance activity
Event Duration Seconds	Seconds between start and end timestamp
Date	Format: *year*-*month*-*day*

Columns of the *product events.csv* and *maintenance events.csv* files. The files contain the timestamps of the start and end of each event, resulting from the refinement in Step 3 of the labelling process.

The *Event Type* column represents the product events from Table 2 or the maintenance events from Table 3, respectively. The timestamps in the *Automatic Timestamp* column correspond to the one-minute granularity timestamps that were automatically generated by the machines.

The refined automatic timestamps from Step 3 of the labelling procedure, as stated in the corresponding description before, are stored in the *Start Timestamp* and *End Timestamp* columns. As a result of using the coffeemakers in an office building, their energy patterns differ considerably between working and non-working days. Therefore, we include an additional CSV-file for each coffeemaker, namely, the *day information.csv*, to provide this information, as shown in Table 9.

**Table 9:** Description of the day and date information files.

Column	Description
Date	Format: *year*-*month*-*day*
WorkingDay	*True* if working day, *False* if not
Weekday	Day of the week

Columns of the day information.csv files that contain the information about working days in Germany and the weekday information for all days in the dataset, for each of the coffeemakers.

In addition to the labels that were generated as a result of the labelling procedure, we also include the raw label files of the product and maintenance events automatically generated by the coffeemakers in the *raw coffee maker logs* subfolders. These files contain the one-minute granularity timestamp, and the columns named *Activity* corresponding to the maintenance events or *Product* for the product events, accordingly.

## Technical Validation

### Signal acquisition

The data collection capabilities of the MEDAL system were thoroughly evaluated concerning the long term measurements presented in the BLOND dataset. In the following subsection, we describe the major characteristics of the hardware technical validation. Additional details can be found in the corresponding data descriptor of the BLOND dataset^16^. We applied the same data sanity checks as the ones implemented for the BLOND dataset collection. The data acquisition unit was used to perform the checks aiming to detect continuity and transmission errors^16^. Furthermore, each file created during a day has a unique sequence number to detect gaps in recordings. To perform offline verification, each HDF5 file included two trigger IDs in its metadata, as presented in Table 6, aiming to ensure a continuous and uninterrupted signal. No discontinuities were presented in CREAM, according to the utilised sequence numbers. MEDAL recorded the signals with the fixed nominal sampling rate of 6400 sps. The actual sampling rate could differ from the nominal one due to minor deviations corresponding to MEDAL’s internal oscillator that was used to control the ADC conversion^16^. Based on the analysis conducted for the BLOND dataset^20^, we analysed the average sampling rate in the data obtained per day. The results of the analysis were in-line with the ones published for the BLOND dataset, indicating that concerning CREAM, the actual sampling rate did not differ from the nominal one. Furthermore, we performed additional sanity checks per file, implemented on the basis of the ones outlined for BLOND. We checked the dataset for completeness, considering the expected number of samples per file, the number of files, and the number of days in the dataset. The analysis results confirm that no gaps were present in the dataset, and the data for all days in the considered period were recorded appropriately. The nominal mains frequency of the electrical network was 50 Hz. We estimated the actual mains frequency based on the voltage signal by selecting the strongest bin in a fast Fourier transform. Deviations from the nominal frequency could indicate malfunctions of the ADC^16^. However, no difference in the frequency was observed. In addition, we implemented the checks to control multiple parameters of the voltage, and current signals, such as the root mean squared (RMS) values. The parameters and their corresponding thresholds are provided in Table 10. The parameters were checked for both current and voltage signal, and as a result, we observed that the tests were passed successfully for all files. In addition to these checks, we validated whether the signals contained flat regions with individual periods consisting only of constant values. The scripts to reproduce the data sanity checks are provided in the CREAM repository^19^, in the *technical validation* subfolder.

**Table 10:** Validated voltage and current parameters.

Parameter *ϕ*	Value range of *ϕ*
Voltage RMS	210 < = *ϕ* < = 240
Voltage mean	0 < = |*ϕ*| < = 5
Voltage crest factor	1.2 < = *ϕ* < = 1.6
Voltage value range	*ϕ* > = 2000
Voltage bandwidth	*ϕ* > = 50
Voltage minimum	−300 < = *ϕ* < = −355
Voltage maximum	300 < = *ϕ* < = 355
Current RMS	0 < = *ϕ* < = 16
Current mean	0 < = |*ϕ*| < = 1
Current crest factor	*ϕ* > = 1.2

Based on the technical validation performed for the MEDAL units in the BLOND dataset^16^, we validated the signal with respect to the parameters listed in this table. The parameter ϕ needs to be within the specified value range to pass the validation.

### Label validation

**Fig. 4:**
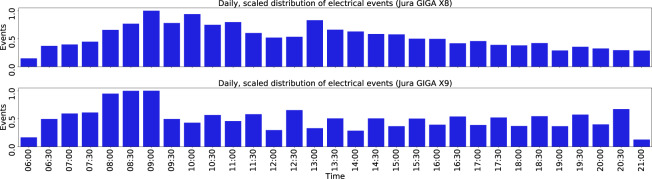
Daily distribution of electrical events. The upper figure shows the scaled event distribution of the *Jura GIGA X8*, whereas the bottom one visualises the event distribution of the *Jura GIGA X9*. Accumulated over all days in the dataset, the distribution of events is balanced with peaks in the morning and after lunch, as expected in the office environment.

We applied several measures to ensure the appropriate labelling quality throughout all steps of the labelling procedure, as outlined in Fig. 2. The main validation component was a double-review of all labelled events. Therefore, all event labels were at least checked independently by two experts. In the case of errors or inaccuracies, the labels were corrected by the expert. During the initial labelling, examples of existing event types were provided to guide the experts through the process. We established the labelling notebooks to prevent labelling errors by introducing pre-labelled event examples to the experts. The electrical event labels from Step 1 are uniformly distributed over the day, and no gaps exist, as shown in Fig. 4 for both coffeemakers.

**Fig. 5:**
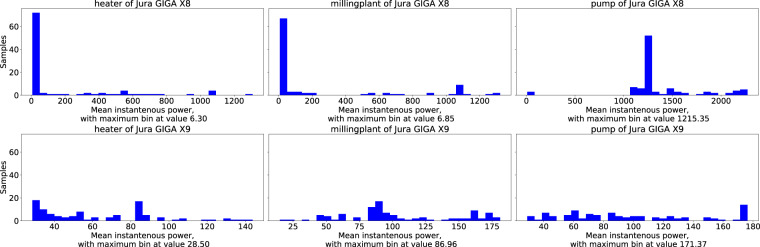
Mean instantaneous power consumed by each main component. We computed the power for a subset of 100 samples per component to address the class imbalance. The first row of the figure contains the components of the *Jura GIGA X8* and the second one the ones of the *Jura GIGA X9*. For the *Jura GIGA X8*, each component has a clear peak power corresponding to the consumption of the majority of events, whereas the *Jura GIGA X9* exhibits more uniform characteristics.

In Step 2 of the labelling process, we assigned each component a subset of the corresponding electrical events. Figure 5 represents the mean instantaneous power consumed by every component, grouped by the corresponding coffeemaker. Due to imbalance in the number of labelled components, we subsampled 100 of them to obtain comparable values. The characteristics of the components differ between the two coffeemakers. The components of the *Jura GIGA X9* are often triggered simultaneously, as the uniform distribution of the instantaneous power consumed shows. This raises the demand for energy disaggregation algorithms to filter out the individual components from overlapping signal segments. In contrast, most of the labelled component patterns in the *Jura GIGA X8* exhibit a uniform power consumption pattern, except for a few outliers. Similar to the *Jura GIGA X9*, deviations from the mean occur when the components are activated simultaneously. Consequently, the signals from individual components superimpose each other.

When analysing the duration of the refined product and maintenance events of the *Jura GIGA X8* obtained at Step 3 of the labelling process, one can see in Table 4 that most of them have a small variation and are labelled with a uniform length in time. In comparison, the duration of the events created by the *Jura GIGA X9* exhibit a higher variation. The deviations can be observed due to various reasons, such as changes in the coffeemaker settings, processes that differ between the coffeemakers or diverging user behaviour. The labelling procedure itself was precise, as confirmed by visual inspection.

## Usage Notes

In the CREAM repository^19^, we provide the code to reproduce the dataset creation and the examples that can be used to facilitate the usage of the dataset. The code is provided in the *source code* folder. All source code is implemented in Python 3. The recorded electrical signals are stored in files in the format of HDF5 files. The HDF5 format is supported by most of the scientific computing libraries, such as Python (h5py/numpy/scipy), MATLAB (h5read), and R (rhdf5). The code snippet in Box 1 shows the usage of the h5py python library for extracting the data.

We provide the relevant metadata in HDF5 attributes within the individual files and the respective filenames, as documented in Table 6. While creating the HDF5 files, we have used the following widely supported filters: gzip compression, shuffle to improve the compression ratio, and Fletcher to add checksums to prevent the data from being corrupted. The repository contains examples of loading and pre-processing the CREAM data. Furthermore, we provide the labelling tools utilised to produce the labels, as outlined in Fig. 2 and as shown in 3. Therefore, the created labels can be reproduced independently. Moreover, the set of existing labels can be extended if necessary. In CREAM, we provide the raw measurements to avoid any bias caused by data pre-processing. Despite that, we recommend applying two pre-processing steps for most of the potential analysis techniques. First, we recommend to calibrate the signals according to the calibration factors provided in the file metadata (see Table 6). Second, we suggest removing any DC-bias by subtracting the mean offset from each mains-cycle in the signal. We provide the implementations for both pre-processing steps in the repository, according to the instructions outlined in the BLOND repository^20,21^.

Box 1: Exemplary usage of the h5py python library for extracting the electrical signals of the coffeemakers and the metadata from the HDF5 data files. In the CREAM repository^19^, we provide pre-built functions for reading and processing the full dataset.

1. import h5py

2.

3. file_path = “file path_of_interest“ # data location

4.

5. # Open the file

6. with h5py. File (file_path, ’r’, driver= ’core’) as f :

7.

8. “““

9. Extract the signal.

10. The file contains the “current1” coffee maker channel, the “current6” noise channel, and the “voltage” data.

11. “““

12. signal = f [“current1”] [:]

13.

14. “““

15. Extract the metadata attributes.

16. The “calibration_factor“ attribute can be replaced by any another one.

17. “““

18. calibration_factor = f [name].attrs [“calibration_factor“]

## Data Availability

The source files for the data collection using the MEDAL measurement units are available in the BLOND data repository^20^. For completeness sake, we have also added these files to the CREAM repository^19^ in the *data collection* folder. This repository contains all the scripts used for the technical validation of the measurement hardware capabilities. The code to reproduce the extraction of the product and maintenance events through the serial maintenance ports of the coffeemakers is available in the coffeemaker project repository provided by Q42^18^. We implemented the data processing, labelling tools, and utility functions in Python 3. The labelling tools were implemented in three Jupyter Notebooks, one corresponding to each step of the labelling pipeline. The individual source files are available in the CREAM repository^19^. All labelling steps can be fully reproduced and extended if necessary, using the supplied tools. Furthermore, we provide the utility class containing all necessary functions for loading and pre-processing the signals.
